# Supplementary material for: HIF-1α Pathway in COVID-19: A Scoping Review of Its Modulation and Related Treatments
Source: Int J Mol Sci. 2025 Apr 28;26(9):4202. doi: 10.3390/ijms26094202 (PMC12071378; doi:10.3390/ijms26094202)
Supplement: Supplementary file 1 [file ijms-26-04202-s001.zip › Suplementt. Material_/INPLASY-Protocol-7042.pdf]

INPLASY

HIF-1α Activation and Related Treatments in COVID-19: A Scoping Review of Inflammatory Mechanisms and Comparisons with Other Hypoxia-Inducing Viral Infections

INPLASY2024110037
doi: 10.37766/inplasy2024.11.0037
Received: 6 November 2024
Published: 7 November 2024

Da Silva, GPF; Matte, R; Menin, MR; Wiedmer, BD.

Corresponding author:
Felipe Paes Gomes da Silva

paes.silva@pucpr.edu.br

Author Affiliation:
Pontifícia Universidade Católica da Paraná - PUCPR.

ADMINISTRATIVE INFORMATION

Support - Authors.

Review Stage at time of this submission - Piloting of the study selection process.

Conflicts of interest - None declared.

INPLASY registration number: INPLASY2024110037

Amendments - This protocol was registered with the International Platform of Registered Systematic Review and Meta-Analysis Protocols (INPLASY) on 7 November 2024 and was last updated on 7 November 2024.

INTRODUCTION

Review question / Objective In both in vitro studies and animal models, as well as in human tissues infected with SARS-CoV-2, how does the activation of HIF-1α differ from its activation in other viral infections that induce hypoxia, particularly regarding the inflammatory response and viral replication? What are the observed effects of treatments that modulate HIF-1α, which have been previously tested in the context of COVID-19?
This review provides an up-to-date synthesis of the current knowledge regarding the molecular mechanisms of HIF-1α in the respiratory tract infected by SARS-CoV-2 and other respiratory viruses, contributing to a deeper understanding of this factor's role in infectious processes and its potential clinical implications.

Background The global emergence of Severe Acute Respiratory Syndrome Coronavirus 2 (SARS-CoV-2) has emphasized the critical need to understand the molecular mechanisms underlying viral infections and host responses (Zhu et al., 2020). Hypoxia, a condition characterized by reduced oxygen availability, is a common feature in severe respiratory infections and significantly influences disease progression (Eltzschig & Carmeliet, 2011). Central to the cellular response to hypoxia is Hypoxia-Inducible Factor 1-alpha (HIF-1α), a transcription factor that regulates the expression of genes involved in metabolism, angiogenesis, and inflammation under low oxygen conditions (Semenza, 2012).
In the context of SARS-CoV-2 infection, HIF-1α has been implicated in modulating the immune response and affecting viral replication dynamics (Codo et al., 2020). The activation of HIF-1α during SARS-CoV-2 infection may lead to distinct

Da Silva et al. INPLASY protocol 2024110037. doi:10.37766/inplasy2024.11.0037 Downloaded from https://inplasy.com/inplasy-2024-11-0037/

inflammatory profiles compared to other hypoxia-inducing viral infections such as influenza and respiratory syncytial virus (Bello & Kitab, 2021). Understanding these differences is crucial for elucidating the pathophysiological mechanisms of COVID-19 and could inform the development of targeted therapeutic strategies.

Moreover, treatments that modulate HIF-1α activity have shown potential in influencing disease outcomes in viral infections (Lee et al., 2019). Investigating the impact of such treatments on SARS-CoV-2 infection could provide valuable insights into novel clinical interventions aimed at mitigating tissue damage and improving patient prognosis.

Rationale A comprehensive exploration of HIF-1α's role in SARS-CoV-2 infection—including its effects on the inflammatory response, viral replication, and the efficacy of HIF-1α-modulating treatments—may contribute to a deeper understanding of COVID-19 pathogenesis and inform future therapeutic approaches.

METHODS

Strategy of data synthesis The data synthesis process will leverage electronic tools to enhance rigor and transparency. Using Rayyan, an online platform for systematic reviews, two independent reviewers will conduct blinded data extraction, ensuring duplicate removal and maintaining blinding and randomization throughout the process. For quantitative analysis, JMP Pro 14.0.0 software (SAS Institute, Cary, NC, USA) will provide robust statistical insights.

In synthesizing data, both paired and unpaired analyses will be implemented. Paired analysis will focus on studies using identical experimental techniques (e.g., immunohistochemistry vs. immunohistochemistry), allowing for direct comparisons, while unpaired analysis will address studies with differing methodologies, assessing consistency across outcomes. Qualitative data will be synthesized thematically to highlight recurring patterns and themes. For quantitative findings, a meta-analysis will be conducted where appropriate, calculating effect sizes with 95% confidence intervals (CI). Statistical measures will be applied to assess heterogeneity, and subgroup analyses will be performed if sufficient data is available.

To assess bias rigorously, the Cochrane Collaboration's tool will be used for randomized clinical trials, and the Newcastle-Ottawa Scale for observational studies, classifying bias as low, medium, or high. Any disagreements between reviewers will be resolved by consulting a third reviewer, with all steps documented to ensure transparency and reproducibility.

Eligibility criteria The eligibility criteria for this scoping review will be carefully articulated to ensure that only studies of the highest relevance and methodological quality are included. To be considered, studies must utilize animal models, in vitro models, or human tissues infected with SARS-CoV-2. Furthermore, these studies should specifically investigate the activation and modulation of HIF-1α within the context of SARS-CoV-2 infection and evaluate treatments that affect HIF-1α activity, including inhibitors, activators, or other modulatory agents relevant to COVID-19.

In addition, this review will incorporate comparative studies that examine SARS-CoV-2 alongside other hypoxia-inducing viral infections, particularly those that assess therapeutic interventions. This will help clarify the distinct roles that HIF-1α may play across different viral infections.

Conversely, stringent exclusion criteria will be applied to eliminate studies that do not focus on respiratory viral infections. This includes studies related to non-respiratory infections that are not connected to SARS-CoV-2 or those that do not explore or discuss the activation or modulation of HIF-1α.

Moreover, the review will be restricted to studies published within a specified timeframe, specifically between 2015 and 2024, and written in English or Portuguese. Selected studies will be limited to specific designs, including longitudinal cohort studies, in vivo and in vitro experimental intervention studies, as well as randomized clinical trials, ensuring a thorough and relevant examination of the subject matter.

Source of evidence screening and selection The selection process for sources in this review will be systematically conducted across multiple stages to ensure methodological rigor and reduce potential biases. Initially, two independent reviewers will conduct a blinded screening of titles and abstracts for all identified studies, with identifying information such as authorship, affiliations, and publication sources concealed to maintain objectivity.

Following this preliminary screening, selected studies will undergo a full-text review, during which the same reviewers will independently assess each study against the predefined eligibility criteria. In instances where discrepancies arise at any stage—whether in the title and abstract screening or the full-text review—these will be addressed through structured discussion between the reviewers. If consensus remains unattainable, a third reviewer

Da Silva et al. INPLASY protocol 2024110037. doi:10.37766/inplasy2024.11.0037 Downloaded from https://inplasy.com/inplasy-2024-11-0037/

will adjudicate to provide a definitive decision. To ensure transparency and reproducibility, all stages of the selection process, including records of disagreements and resolutions, will be meticulously documented in an Excel spreadsheet.

Data management The data management process for this review will be rigorously structured to uphold consistency, transparency, and reproducibility throughout. Rayyan, a specialized online platform for systematic reviews, will be employed to facilitate the initial screening and data extraction phases, enabling blinded extraction by two independent reviewers, duplicate removal, and randomized study assignment.

Data from eligible studies will be systematically extracted using a pre-designed data extraction form, ensuring consistency in capturing key study variables. Extracted data will include study characteristics such as authorship, publication year, study design, sample size, and core findings related to HIF-1α activation, modulation, and outcomes in SARS-CoV-2 infection and other hypoxia-inducing viral infections.

Following extraction, data will be transferred into an Excel spreadsheet to support organization and to streamline the synthesis and analysis phases. This spreadsheet will serve as a central repository, systematically documenting all extracted information and tracking decisions made throughout the review process, including records of any disagreements and their resolutions. Quantitative data analysis, where applicable, will be conducted using JMP Pro 14.0.0 software (SAS Institute, Cary, NC, USA), facilitating robust statistical insights.

To ensure data integrity and security, all digital files will be stored on a secure, password-protected server, with access restricted to the review team. Regular backups will be performed to mitigate data loss risks. Each step in the data management process will be meticulously documented, ensuring auditability and reproducibility for future verification.

Language restriction English and Portuguese.

Country(ies) involved Brazil and China.

Keywords Hypoxia-Inducible Factor 1, alpha Subunit; Hypoxia; COVID-19; Virus Diseases; Inflammation; Virus Replication.

Dissemination plans To disseminate the findings, the group has agreed to submit the article for peer review in the Special Issue of the International Journal of Molecular Sciences, published by MDPI.

Contributions of each author

Author 1 - Felipe Paes Gomes da Silva.
Email: paes.silva@pucpr.edu.br
Author 2 - Rafael Matte.
Email: rafael.matte@pucpr.edu.br
Author 3 - David Batista Wiedmer.
Author 4 - Rafaela Makiak Menin.

Da Silva et al. INPLASY protocol 2024110037. doi:10.37766/inplasy2024.11.0037 Downloaded from https://inplasy.com/inplasy-2024-11-0037/
