# Supplementary material for: HIF-1α Pathway in COVID-19: A Scoping Review of Its Modulation and Related Treatments
Source: Int J Mol Sci. 2025 Apr 28;26(9):4202. doi: 10.3390/ijms26094202 (PMC12071378; doi:10.3390/ijms26094202)
Supplement: Supplementary file 1 [file ijms-26-04202-s001.zip › Suplementt. Material_/SUPPLEMENTARY MATERIAL - MeSH TERMS.pdf]

SUPPLEMENTARY MATERIAL -
Terms Used to Search in PubMed and Scopus Using Boolean Operators

1) **MeSH PubMed**

(Hypoxia-Inducible Factor 1) AND (COVID-19)
(Hypoxia-Inducible Factor 1) AND (COVID-19) AND (Treatment)
(Hypoxia-Inducible Factor 1) AND (SARS-CoV-2)
(Hypoxia-Inducible Factor 1) AND (SARS-CoV-2) AND (Treatment)
(Hypoxia-Inducible Factor 1) AND (SARS-CoV-2) AND (Post-Acute COVID-19 Syndrome)
(Hypoxia-Inducible Factor 1) AND (COVID-19) AND (PACS)
(HIF) AND (COVID-19) AND (Post-Acute COVID-19 Syndrome)
(HIF) AND (SARS-CoV-2) AND (Post-Acute COVID-19 Syndrome)
(HIF) AND (SARS-CoV-2)
(HIF) AND (COVID-19)
(HIF) AND (SARS-CoV-2) AND (treatment)
(HIF) AND (COVID-19) AND (treatment)
(HIF) AND (Long COVID)

2) **MeSH Elsevier**

(HIF) AND (COVID-19)
(HIF) AND (SARS-CoV-2)
